# One community, multiple perspectives: ATEMPO 12 as a model for adaptive early psychosis care

**DOI:** 10.1192/j.eurpsy.2026.10259

**Published:** 2026-07-31

**Authors:** A. Compaired Sánchez

**Affiliations:** Psychiatry, 12 de Octubre University Hospital, Madrid, Spain

## Abstract

**Abstract:**

Early intervention in psychosis must extend beyond reducing duration of untreated psychosis; it requires adaptive systems capable of addressing clinical complexity within the socioeconomic realities of the communities they serve. ATEMPO 12, the First Episode Psychosis (FEP) program at Hospital Universitario 12 de Octubre (Madrid), has evolved over 15 years as a community-embedded, interdisciplinary model serving a predominantly low-income, culturally diverse urban population characterized by high substance use prevalence, migration-related stressors, and structural barriers to sustained care.

The program integrates psychiatry, clinical psychology, nursing, and social work within a flexible framework that connects acute inpatient units, rehabilitation and addiction services, and in-house specialized programs for alcohol-use disorder, treatment-resistant depression, resistant obsessive-compulsive disorder, and eating disorders. Community mental health centers—formally integrated into the program and offering extended afternoon schedules—facilitate additional outpatient follow-up or parallel care pathways when hospital attendance is limited by economic or social constraints. This stepped-care ecosystem enables early stabilization while preserving longitudinal continuity.

Clinical work is grounded in precision psychopathology: detailed phenomenological assessment, attention to personality structure, and rigorous differential diagnosis, including neurodevelopmental comorbidities. Psychotherapy follows a modular, stage-sensitive design combining psychoeducation, cognitive behavioral therapy for psychosis, metacognitive interventions, positive psychology strategies, family-based programs, and neuropsychological evaluation when indicated.

Psychopharmacological management emphasizes shared decision-making and fine-grained personalization, integrating pharmacokinetic principles, receptor-informed strategies, subjective experience, and functional outcomes. Long-acting injectable antipsychotics are considered early in selected cases with adherence vulnerability, while a structured clozapine pathway and access to repetitive transcranial magnetic stimulation (rTMS) and electroconvulsive therapy support treatment-resistant presentations.

Drawing on a large real-world cohort (2009–present), including patients with affective and substance-use comorbidity, ATEMPO 12 illustrates how interdisciplinary early psychosis systems can reconcile scientific rigor with social complexity. The model defends that early psychosis care must be flexible, culturally responsive, and pharmacologically sophisticated—bridging neuroscience, psychotherapy, and service design to optimize long-term trajectories.

**Disclosure of Interest:**

None Declared

